# 4-[5-(Furan-2-yl)-3-trifluoro­methyl-1*H*-pyrazol-1-yl]benzene­sulfonamide

**DOI:** 10.1107/S1600536812011920

**Published:** 2012-03-24

**Authors:** Abdullah M. Asiri, Hassan M. Faidallah, Khalid A. Alamry, Seik Weng Ng, Edward R. T. Tiekink

**Affiliations:** a Chemistry Department, Faculty of Science, King Abdulaziz University, PO Box 80203, Jeddah, Saudi Arabia; b The Center of Excellence for Advanced Materials Research, King Abdulaziz University, Jeddah, PO Box 80203, Saudi Arabia; c Department of Chemistry, University of Malaya, 50603 Kuala Lumpur, Malaysia

## Abstract

In the title compound, C_14_H_10_F_3_N_3_O_3_S, there are significant twists in the mol­ecule, as seen in the values of the dihedral angles between the pyrazole ring and each of the furan [31.1 (2)°] and benzene rings [55.58 (10)°]. The amino N atom occupies a position almost normal to the benzene ring [N—S—C_ar_—C_ar_ (ar = aromatic) torsion angle = 83.70 (19)°]. One amino H atom forms a hydrogen bond to the tricoordinate pyrazole N atom and the other inter­acts with a sulfonamide O atom, forming a supra­molecular chain along [010]. The chains are consolidated into a supra­molecular layers *via* C—H⋯O inter­actions involving the second sulfonamide O atom; layers stack along [10-1]. The furan ring was found to be disordered over two diagonally opposite orientations of equal occupancy.

## Related literature

For background to the biological applications of sulfonamides, see: Croitoru *et al.* (2004 ▶); Dogruer *et al.* (2010 ▶). For the biological efficacy of F and CF_3_ in medicinal chemistry, see: Fokin & Kolomiyets (1988 ▶); Bonacorso *et al.* (2006 ▶). For related structures, see: Asiri *et al.* (2011 ▶, 2012 ▶).
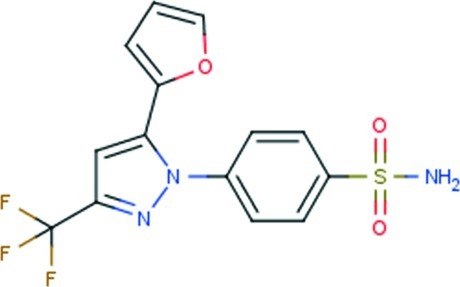


## Experimental

### 

#### Crystal data

C_14_H_10_F_3_N_3_O_3_S
*M* *_r_* = 357.31
Monoclinic, 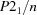

*a* = 16.0536 (13) Å
*b* = 4.8173 (4) Å
*c* = 20.6202 (15) Å
β = 110.728 (8)°
*V* = 1491.4 (2) Å^3^
*Z* = 4
Mo *K*α radiation
μ = 0.27 mm^−1^
*T* = 100 K
0.25 × 0.10 × 0.05 mm

#### Data collection

Agilent SuperNova Dual diffractometer with an Atlas detector
Absorption correction: multi-scan (*CrysAlis PRO*; Agilent, 2011 ▶) *T*_min_ = 0.935, *T*_max_ = 0.987
6503 measured reflections
3431 independent reflections
2400 reflections with *I* > 2σ(*I*)
*R*_int_ = 0.051

#### Refinement

*R*[*F* ^2^ > 2σ(*F* ^2^)] = 0.069
*wR*(*F* ^2^) = 0.189
*S* = 1.06
3431 reflections
210 parameters
33 restraints
H atoms treated by a mixture of independent and constrained refinement
Δρ_max_ = 0.57 e Å^−3^
Δρ_min_ = −0.63 e Å^−3^

### 

Data collection: *CrysAlis PRO* (Agilent, 2011 ▶); cell refinement: *CrysAlis PRO*; data reduction: *CrysAlis PRO*; program(s) used to solve structure: *SHELXS97* (Sheldrick, 2008 ▶); program(s) used to refine structure: *SHELXL97* (Sheldrick, 2008 ▶); molecular graphics: *X-SEED* (Barbour, 2001 ▶) and *DIAMOND* (Brandenburg, 2006 ▶); software used to prepare material for publication: *publCIF* (Westrip, 2010 ▶).

## Supplementary Material

Crystal structure: contains datablock(s) global, I. DOI: 10.1107/S1600536812011920/hb6693sup1.cif

Structure factors: contains datablock(s) I. DOI: 10.1107/S1600536812011920/hb6693Isup2.hkl

Supplementary material file. DOI: 10.1107/S1600536812011920/hb6693Isup3.cml

Additional supplementary materials:  crystallographic information; 3D view; checkCIF report

## Figures and Tables

**Table 1:** Hydrogen-bond geometry (Å, °)

*D*—H⋯*A*	*D*—H	H⋯*A*	*D*⋯*A*	*D*—H⋯*A*
N3—H1⋯O3^i^	0.88 (1)	2.00 (2)	2.830 (4)	158 (3)
N3—H2⋯N1^ii^	0.88 (1)	2.17 (1)	3.032 (4)	170 (4)
C8—H8⋯O2^iii^	0.95	2.36	3.185 (7)	145
C10—H10⋯O2^iv^	0.95	2.44	3.092 (4)	126

Symmetry codes: (i) 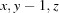; (ii) 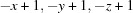; (iii) 
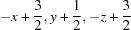
; (iv) 
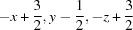
.

**Fig. 1.**
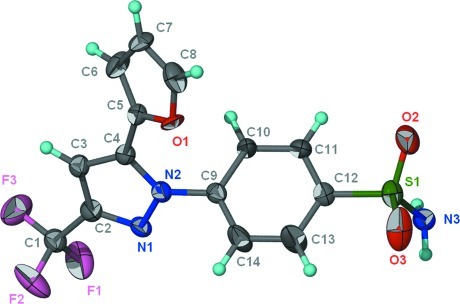
The molecular structure of (I) showing displacement ellipsoids at the 70% probability level. Only one orientation of the disordered furanyl ring is shown.

**Fig. 2.**
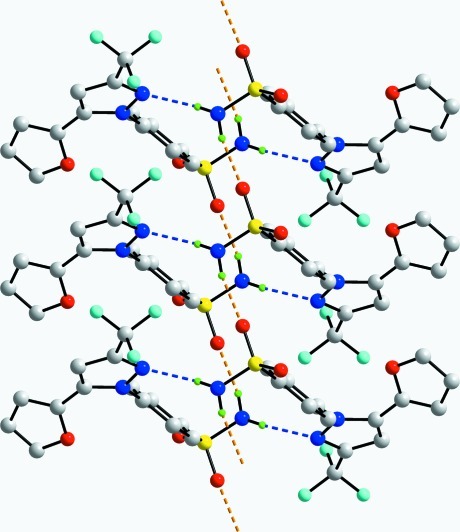
Supramolecular chain along [010] in (I) sustained by N—H···N and N—H···O hydrogen bonds shown as blue and orange dashed lines, respectively. Hydrogen atoms not participating in hydrogen-bonding interactions have been omitted for reasons of clarity.

**Fig. 3.**
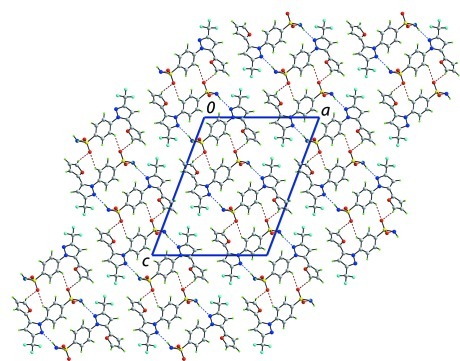
A view in projection down the b axis of the unit-cell contents of (I). The N—H···N, N—H···O and C—H···O interactions are shown as orange, blue and brown dashed lines, respectively.

## supplementary crystallographic information

### Comment

The title CF_3_-derivatized sulfonamide (I), was investigated. in connection with on-going studies of sulfonamides, both biological (Croitoru *et al.*, 2004; Dogruer *et al.*, 2010) and crystallographic (Asiri *et al.*, 2011; Asiri *et al.*, 2012). In particular, fluoride in the form of a trifluoromethyl group, which has long been recognized in medicinal chemistry for its ability to alter the physico-chemical and biological characteristics of molecules (Fokin & Kolomiyets, 1988; Bonacorso *et al.*, 2006), is featured in the new molecule to promote enhanced biological properties.

In (I), Fig. 1, the dihedral angle of 31.1 (2)° between the furanyl and pyrazole rings indicates a significant twist between these rings. Similarly, the dihedral angle of 55.58 (10)° between the pyrazole ring and the benzene ring to which it is connected indicates a significant twist. The amino-N3 atom occupies a position almost normal to the benzene ring, forming a N3—S1—C12—C13 torsion angle of 83.70 (19)°. This allows the participation of both N—H atoms in hydrogen bonding interactions.

One amino-H atom forms a hydrogen bond to the pyrazole-N2 atom of a centrosymmetrically related molecule to form an 18-membered {···HNSC_4_NN}_2_ synthon, Table 1. These are connected into a supramolecular chain *via* a *N*—*H*···*O*(sulfonamide) hydrogen bonding, Fig. 2 and Table 1. The second sulfonamide-O2 atom forms two C—H···O interactions, Table 1, to consolidate the chains into a supramolecular layers. No specific intermolecular interactions occur between the layers that stack along [1 0 1], Fig. 3.

### Experimental

A solution of 4,4,4-trifluoro-1-phenyl-1,3-butanedione (2.16 g, 0.01 mmol) in ethanol (50 ml) was refluxed with 4-hydrazinobenzenesulfonamide hydrochloride (2.2 g, 0.01 mmol) for 4 h, concentrated and cooled. The precipitated crude product was filtered and recrystallized from ethanol as colourless prisms. Yield: 74%. *M*.pt: 467–468 K.

### Refinement

Carbon-bound H-atoms were placed in calculated positions [C—H = 0.95 Å; *U*_iso_(H) = 1.2*U*_eq_(C)] and were included in the refinement in the riding model approximation. The N—H atoms were located in a difference Fourier map, and were refined with a distance restraint of N—H = 0.88±0.01 Å; their *U*_iso_ values were refined.

The furyl ring is disordered over two positions; the disorder could not be refined, and was assumed as a 1:1 type of disorder. The ring was refined as a rigid pentagon of 1.35 Å sides. The *U_ij_* values of C6' was equated to those of O6 (as well as the O1'/C6, C5'/C5, C6'/O1, C7'/C8 and C8'/C7 pairs). The benzene ring was refined as a rigid hexagon of 1.39 Å sides.

Owing to poor agreement, the (8 0 10) reflection was omitted from the final cycles of refinement.

### Crystal data

**Table:**

C_14_H_10_F_3_N_3_O_3_S	*F*(000) = 728
*M**_r_* = 357.31	*D*_x_ = 1.591 Mg m^−^^3^
Monoclinic, *P*2_1_/*n*	Mo *K*α radiation, λ = 0.71073 Å
Hall symbol: -P 2yn	Cell parameters from 1899 reflections
*a* = 16.0536 (13) Å	θ = 2.5–27.5°
*b* = 4.8173 (4) Å	µ = 0.27 mm^−^^1^
*c* = 20.6202 (15) Å	*T* = 100 K
β = 110.728 (8)°	Prism, colourless
*V* = 1491.4 (2) Å^3^	0.25 × 0.10 × 0.05 mm
*Z* = 4	

### Data collection

**Table:**

Agilent SuperNova Dual diffractometer with an Atlas detector	3431 independent reflections
Radiation source: SuperNova (Mo) X-ray Source	2400 reflections with *I* > 2σ(*I*)
Mirror monochromator	*R*_int_ = 0.051
Detector resolution: 10.4041 pixels mm^-1^	θ_max_ = 27.6°, θ_min_ = 2.7°
ω scan	*h* = −20→15
Absorption correction: multi-scan (*CrysAlis PRO*; Agilent, 2011)	*k* = −6→6
*T*_min_ = 0.935, *T*_max_ = 0.987	*l* = −26→25
6503 measured reflections	

### Refinement

**Table:**

Refinement on *F*^2^	Primary atom site location: structure-invariant direct methods
Least-squares matrix: full	Secondary atom site location: difference Fourier map
*R*[*F*^2^ > 2σ(*F*^2^)] = 0.069	Hydrogen site location: inferred from neighbouring sites
*wR*(*F*^2^) = 0.189	H atoms treated by a mixture of independent and constrained refinement
*S* = 1.06	*w* = 1/[σ^2^(*F*_o_^2^) + (0.0768*P*)^2^ + 1.5964*P*] where *P* = (*F*_o_^2^ + 2*F*_c_^2^)/3
3431 reflections	(Δ/σ)_max_ = 0.001
210 parameters	Δρ_max_ = 0.57 e Å^−^^3^
33 restraints	Δρ_min_ = −0.63 e Å^−^^3^

### Fractional atomic coordinates and isotropic or equivalent isotropic displacement parameters (Å^2^)

**Table:**

	*x*	*y*	*z*	*U*_iso_*/*U*_eq_	Occ. (<1)
S1	0.55947 (6)	0.78623 (17)	0.68278 (5)	0.0271 (3)	
F1	0.67465 (17)	−0.1684 (5)	0.34173 (13)	0.0506 (7)	
F2	0.7248 (2)	0.1593 (5)	0.29748 (11)	0.0559 (7)	
F3	0.81166 (17)	−0.1668 (6)	0.35146 (14)	0.0605 (8)	
O1	0.8796 (3)	0.7601 (14)	0.6001 (3)	0.0229 (13)	0.50
C5	0.9023 (5)	0.5273 (19)	0.5738 (4)	0.0223 (8)	0.50
C6	0.9876 (5)	0.4658 (18)	0.6122 (5)	0.0330 (13)	0.50
H6	1.0207	0.3124	0.6052	0.040*	0.50
C7	1.0177 (3)	0.6606 (15)	0.6621 (3)	0.0298 (17)	0.50
H7	1.0757	0.6683	0.6965	0.036*	0.50
C8	0.9510 (4)	0.8425 (11)	0.6547 (3)	0.035 (2)	0.50
H8	0.9537	1.0006	0.6829	0.042*	0.50
O1'	0.9946 (4)	0.4216 (15)	0.5961 (3)	0.0330 (13)	0.50
C5'	0.9085 (4)	0.501 (2)	0.5745 (4)	0.0223 (8)	0.50
C6'	0.9012 (4)	0.7080 (18)	0.6164 (4)	0.0229 (13)	0.50
H6'	0.8480	0.8029	0.6130	0.028*	0.50
C7'	0.9829 (4)	0.7561 (13)	0.6638 (3)	0.035 (2)	0.50
H7'	0.9972	0.8908	0.6998	0.042*	0.50
C8'	1.0406 (3)	0.5791 (14)	0.6513 (3)	0.0298 (17)	0.50
H8'	1.1027	0.5675	0.6768	0.036*	0.50
O2	0.61854 (18)	0.7401 (7)	0.75194 (14)	0.0456 (8)	
O3	0.5296 (2)	1.0621 (5)	0.65989 (17)	0.0512 (9)	
N1	0.70506 (19)	0.2448 (6)	0.44062 (14)	0.0242 (6)	
N2	0.75031 (18)	0.3840 (6)	0.50020 (14)	0.0233 (6)	
N3	0.4716 (2)	0.6093 (6)	0.67061 (15)	0.0246 (6)	
C1	0.7446 (2)	−0.0041 (8)	0.35257 (17)	0.0289 (8)	
C2	0.7692 (2)	0.1556 (7)	0.41875 (17)	0.0263 (7)	
C3	0.8543 (2)	0.2312 (7)	0.46230 (17)	0.0254 (7)	
H3	0.9094	0.1894	0.4571	0.030*	
C4	0.8404 (2)	0.3810 (7)	0.51480 (17)	0.0240 (7)	
C9	0.70348 (15)	0.4776 (5)	0.54273 (10)	0.0215 (7)	
C10	0.72944 (14)	0.3863 (5)	0.61097 (11)	0.0441 (11)	
H10	0.7778	0.2604	0.6285	0.053*	
C11	0.68462 (16)	0.4790 (6)	0.65348 (8)	0.0384 (10)	
H11	0.7024	0.4165	0.7001	0.046*	
C12	0.61384 (15)	0.6630 (5)	0.62776 (10)	0.0238 (7)	
C13	0.58788 (17)	0.7544 (5)	0.55953 (12)	0.0555 (14)	
H13	0.5395	0.8802	0.5419	0.067*	
C14	0.63270 (18)	0.6617 (5)	0.51701 (9)	0.0457 (11)	
H14	0.6150	0.7242	0.4704	0.055*	
H1	0.481 (2)	0.430 (3)	0.6746 (19)	0.032 (10)*	
H2	0.4245 (17)	0.659 (8)	0.6352 (14)	0.041 (12)*	

### Atomic displacement parameters (Å^2^)

**Table:**

	*U*^11^	*U*^22^	*U*^33^	*U*^12^	*U*^13^	*U*^23^
S1	0.0345 (5)	0.0213 (4)	0.0295 (5)	−0.0030 (4)	0.0161 (4)	−0.0064 (3)
F1	0.0543 (16)	0.0558 (15)	0.0475 (15)	−0.0290 (13)	0.0251 (13)	−0.0239 (12)
F2	0.094 (2)	0.0472 (14)	0.0228 (12)	−0.0098 (14)	0.0162 (13)	−0.0029 (11)
F3	0.0408 (15)	0.0808 (19)	0.0521 (16)	0.0169 (13)	0.0069 (12)	−0.0314 (14)
O1	0.012 (3)	0.026 (3)	0.025 (3)	0.009 (2)	−0.001 (2)	−0.002 (3)
C5	0.0209 (18)	0.024 (2)	0.0233 (16)	0.0009 (15)	0.0097 (14)	0.0001 (15)
C6	0.0217 (18)	0.035 (3)	0.042 (3)	0.0019 (17)	0.012 (2)	−0.011 (2)
C7	0.011 (3)	0.042 (5)	0.032 (3)	−0.003 (3)	0.002 (2)	0.001 (3)
C8	0.048 (5)	0.032 (4)	0.030 (3)	−0.008 (4)	0.020 (3)	−0.008 (3)
O1'	0.0217 (18)	0.035 (3)	0.042 (3)	0.0019 (17)	0.012 (2)	−0.011 (2)
C5'	0.0209 (18)	0.024 (2)	0.0233 (16)	0.0009 (15)	0.0097 (14)	0.0001 (15)
C6'	0.012 (3)	0.026 (3)	0.025 (3)	0.009 (2)	−0.001 (2)	−0.002 (3)
C7'	0.048 (5)	0.032 (4)	0.030 (3)	−0.008 (4)	0.020 (3)	−0.008 (3)
C8'	0.011 (3)	0.042 (5)	0.032 (3)	−0.003 (3)	0.002 (2)	0.001 (3)
O2	0.0350 (15)	0.077 (2)	0.0248 (14)	−0.0097 (14)	0.0100 (12)	−0.0201 (14)
O3	0.082 (2)	0.0163 (13)	0.079 (2)	0.0030 (13)	0.058 (2)	−0.0024 (13)
N1	0.0237 (14)	0.0303 (15)	0.0172 (14)	−0.0032 (12)	0.0054 (11)	−0.0016 (11)
N2	0.0215 (14)	0.0286 (14)	0.0202 (14)	−0.0026 (12)	0.0079 (12)	−0.0006 (11)
N3	0.0285 (16)	0.0206 (14)	0.0247 (16)	0.0029 (12)	0.0094 (13)	0.0014 (12)
C1	0.0273 (18)	0.0350 (19)	0.0242 (17)	−0.0031 (15)	0.0089 (15)	−0.0037 (15)
C2	0.0258 (18)	0.0289 (17)	0.0242 (18)	−0.0009 (14)	0.0090 (15)	−0.0004 (14)
C3	0.0226 (17)	0.0306 (18)	0.0243 (17)	−0.0025 (14)	0.0100 (14)	−0.0011 (14)
C4	0.0199 (17)	0.0304 (17)	0.0224 (17)	−0.0006 (13)	0.0084 (14)	0.0028 (14)
C9	0.0215 (16)	0.0230 (16)	0.0200 (16)	−0.0025 (13)	0.0073 (13)	0.0004 (13)
C10	0.033 (2)	0.078 (3)	0.0251 (19)	0.031 (2)	0.0155 (17)	0.020 (2)
C11	0.027 (2)	0.066 (3)	0.0211 (18)	0.0152 (19)	0.0077 (15)	0.0105 (18)
C12	0.0291 (18)	0.0192 (15)	0.0249 (18)	−0.0011 (13)	0.0118 (15)	−0.0011 (13)
C13	0.082 (4)	0.057 (3)	0.036 (2)	0.054 (3)	0.032 (2)	0.024 (2)
C14	0.071 (3)	0.046 (2)	0.028 (2)	0.033 (2)	0.026 (2)	0.0176 (18)

### Geometric parameters (Å, º)

**Table:**

S1—O2	1.423 (3)	C7'—H7'	0.9500
S1—O3	1.435 (3)	C8'—H8'	0.9500
S1—N3	1.590 (3)	N1—C2	1.334 (4)
S1—C12	1.7614 (17)	N1—N2	1.362 (4)
F1—C1	1.327 (4)	N2—C4	1.369 (4)
F2—C1	1.325 (4)	N2—C9	1.416 (3)
F3—C1	1.338 (4)	N3—H1	0.878 (10)
O1—C5	1.3500	N3—H2	0.878 (10)
O1—C8	1.3500	C1—C2	1.493 (5)
C5—C6	1.3500	C2—C3	1.391 (5)
C5—C4	1.452 (5)	C3—C4	1.383 (5)
C6—C7	1.3500	C3—H3	0.9500
C6—H6	0.9500	C9—C10	1.3900
C7—C8	1.3500	C9—C14	1.3900
C7—H7	0.9500	C10—C11	1.3900
C8—H8	0.9500	C10—H10	0.9500
O1'—C5'	1.3500	C11—C12	1.3900
O1'—C8'	1.3500	C11—H11	0.9500
C5'—C6'	1.3500	C12—C13	1.3900
C5'—C4	1.446 (5)	C13—C14	1.3900
C6'—C7'	1.3500	C13—H13	0.9500
C6'—H6'	0.9500	C14—H14	0.9500
C7'—C8'	1.3500		
			
O2—S1—O3	120.06 (19)	S1—N3—H2	116 (3)
O2—S1—N3	108.29 (17)	H1—N3—H2	115 (4)
O3—S1—N3	105.67 (18)	F2—C1—F1	106.1 (3)
O2—S1—C12	106.65 (14)	F2—C1—F3	106.5 (3)
O3—S1—C12	106.52 (14)	F1—C1—F3	106.6 (3)
N3—S1—C12	109.37 (14)	F2—C1—C2	112.5 (3)
C5—O1—C8	108.0	F1—C1—C2	113.3 (3)
C6—C5—O1	108.0	F3—C1—C2	111.3 (3)
C6—C5—C4	129.5 (5)	N1—C2—C3	113.4 (3)
O1—C5—C4	122.5 (5)	N1—C2—C1	119.3 (3)
C5—C6—C7	108.0	C3—C2—C1	127.4 (3)
C5—C6—H6	126.0	C4—C3—C2	104.4 (3)
C7—C6—H6	126.0	C4—C3—H3	127.8
C8—C7—C6	108.0	C2—C3—H3	127.8
C8—C7—H7	126.0	N2—C4—C3	106.5 (3)
C6—C7—H7	126.0	N2—C4—C5'	127.1 (4)
C7—C8—O1	108.0	C3—C4—C5'	126.3 (4)
C7—C8—H8	126.0	N2—C4—C5	122.4 (4)
O1—C8—H8	126.0	C3—C4—C5	131.1 (5)
C5'—O1'—C8'	108.0	C10—C9—C14	120.0
O1'—C5'—C6'	108.0	C10—C9—N2	119.44 (17)
O1'—C5'—C4	122.9 (5)	C14—C9—N2	120.56 (17)
C6'—C5'—C4	129.0 (5)	C9—C10—C11	120.0
C7'—C6'—C5'	108.0	C9—C10—H10	120.0
C7'—C6'—H6'	126.0	C11—C10—H10	120.0
C5'—C6'—H6'	126.0	C12—C11—C10	120.0
C8'—C7'—C6'	108.0	C12—C11—H11	120.0
C8'—C7'—H7'	126.0	C10—C11—H11	120.0
C6'—C7'—H7'	126.0	C13—C12—C11	120.0
C7'—C8'—O1'	108.0	C13—C12—S1	120.39 (12)
C7'—C8'—H8'	126.0	C11—C12—S1	119.58 (12)
O1'—C8'—H8'	126.0	C12—C13—C14	120.0
C2—N1—N2	103.6 (3)	C12—C13—H13	120.0
N1—N2—C4	112.2 (3)	C14—C13—H13	120.0
N1—N2—C9	119.0 (3)	C13—C14—C9	120.0
C4—N2—C9	128.2 (3)	C13—C14—H14	120.0
S1—N3—H1	113 (3)	C9—C14—H14	120.0
			
C8—O1—C5—C6	0.0	C2—C3—C4—C5	−176.4 (7)
C8—O1—C5—C4	179.3 (12)	O1'—C5'—C4—N2	159.5 (5)
O1—C5—C6—C7	0.0	C6'—C5'—C4—N2	−22.8 (12)
C4—C5—C6—C7	−179.3 (13)	O1'—C5'—C4—C3	−18.1 (12)
C5—C6—C7—C8	0.0	C6'—C5'—C4—C3	159.7 (6)
C6—C7—C8—O1	0.0	O1'—C5'—C4—C5	−159 (10)
C5—O1—C8—C7	0.0	C6'—C5'—C4—C5	18 (9)
C8'—O1'—C5'—C6'	0.0	C6—C5—C4—N2	150.0 (6)
C8'—O1'—C5'—C4	178.2 (12)	O1—C5—C4—N2	−29.1 (12)
O1'—C5'—C6'—C7'	0.0	C6—C5—C4—C3	−33.5 (12)
C4—C5'—C6'—C7'	−178.0 (13)	O1—C5—C4—C3	147.3 (6)
C5'—C6'—C7'—C8'	0.0	C6—C5—C4—C5'	8 (9)
C6'—C7'—C8'—O1'	0.0	O1—C5—C4—C5'	−171 (10)
C5'—O1'—C8'—C7'	0.0	N1—N2—C9—C10	121.0 (3)
C2—N1—N2—C4	0.0 (3)	C4—N2—C9—C10	−49.5 (4)
C2—N1—N2—C9	−171.9 (3)	N1—N2—C9—C14	−59.7 (3)
N2—N1—C2—C3	0.3 (4)	C4—N2—C9—C14	129.8 (3)
N2—N1—C2—C1	−178.7 (3)	C14—C9—C10—C11	0.0
F2—C1—C2—N1	85.1 (4)	N2—C9—C10—C11	179.3 (2)
F1—C1—C2—N1	−35.3 (5)	C9—C10—C11—C12	0.0
F3—C1—C2—N1	−155.4 (3)	C10—C11—C12—C13	0.0
F2—C1—C2—C3	−93.8 (4)	C10—C11—C12—S1	−178.32 (19)
F1—C1—C2—C3	145.8 (4)	O2—S1—C12—C13	−159.41 (19)
F3—C1—C2—C3	25.7 (5)	O3—S1—C12—C13	−30.1 (2)
N1—C2—C3—C4	−0.5 (4)	N3—S1—C12—C13	83.70 (19)
C1—C2—C3—C4	178.4 (3)	O2—S1—C12—C11	18.9 (2)
N1—N2—C4—C3	−0.3 (4)	O3—S1—C12—C11	148.3 (2)
C9—N2—C4—C3	170.6 (3)	N3—S1—C12—C11	−98.0 (2)
N1—N2—C4—C5'	−178.3 (7)	C11—C12—C13—C14	0.0
C9—N2—C4—C5'	−7.3 (8)	S1—C12—C13—C14	178.31 (19)
N1—N2—C4—C5	176.9 (6)	C12—C13—C14—C9	0.0
C9—N2—C4—C5	−12.1 (8)	C10—C9—C14—C13	0.0
C2—C3—C4—N2	0.5 (4)	N2—C9—C14—C13	−179.3 (2)
C2—C3—C4—C5'	178.4 (7)		

### Hydrogen-bond geometry (Å, º)

**Table:**

*D*—H···*A*	*D*—H	H···*A*	*D*···*A*	*D*—H···*A*
N3—H1···O3^i^	0.88 (1)	2.00 (2)	2.830 (4)	158 (3)
N3—H2···N1^ii^	0.88 (1)	2.17 (1)	3.032 (4)	170 (4)
C8—H8···O2^iii^	0.95	2.36	3.185 (7)	145
C10—H10···O2^iv^	0.95	2.44	3.092 (4)	126

Symmetry codes: (i) *x*, *y*−1, *z*; (ii) −*x*+1, −*y*+1, −*z*+1; (iii) −*x*+3/2, *y*+1/2, −*z*+3/2; (iv) −*x*+3/2, *y*−1/2, −*z*+3/2.

## References

[bb1] Agilent (2011). *CrysAlis PRO* Agilent Technologies, Yarnton, England.

[bb2] Asiri, A. M., Al-Youbi, A. O., Faidallah, H. M., Ng, S. W. & Tiekink, E. R. T. (2011). *Acta Cryst.* E**67**, o2424. 10.1107/S1600536811033435 PMC3200877 22059006

[bb3] Asiri, A. M., Faidallah, H. M., Ng, S. W. & Tiekink, E. R. T. (2012). *Acta Cryst.* E**68**, o762–o763. 10.1107/S1600536812006502 PMC3295527 22412638

[bb4] Barbour, L. J. (2001). *J. Supramol. Chem.* **1**, 189–191.

[bb5] Bonacorso, H. G., Wentz, A. P., Lourega, R. V., Cechinel, C. A., Moraes, T. S., Coelho, H. S., Zanatta, N., Martins, M. A. P., Hoerner, M. & Alves, S. H. (2006). *J. Fluorine Chem.* **127**, 1066–1072.

[bb6] Brandenburg, K. (2006). *DIAMOND* Crystal Impact GbR, Bonn, Germany.

[bb7] Croitoru, M., Pintilie, L., Tanase, C., Caproiu, M. T. & Draghici, C. (2004). *Rev. Chem. (Bucharest)*, **55**, 993–997.

[bb8] Dogruer, D. S., Urlu, S., Onkol, T., Ozcelik, B. & Sahin, M. F. (2010). *Turk. J. Chem.* **34**, 57–65.

[bb9] Fokin, A. V. & Kolomiyets, A. F. (1988). *J. Fluorine Chem.* **40**, 247–259.

[bb10] Sheldrick, G. M. (2008). *Acta Cryst.* A**64**, 112–122. 10.1107/S0108767307043930 18156677

[bb11] Westrip, S. P. (2010). *J. Appl. Cryst.* **43**, 920–925.

